# Cerebrospinal fluid biomarkers for Alzheimer's and vascular disease vary by age, gender, and *APOE* genotype in cognitively normal adults

**DOI:** 10.1186/s13195-017-0271-9

**Published:** 2017-07-03

**Authors:** Ge Li, Jane B. Shofer, Eric C. Petrie, Chang-En Yu, Charles W. Wilkinson, Dianne P. Figlewicz, Andrew Shutes-David, Jing Zhang, Thomas J. Montine, Murray A. Raskind, Joseph F. Quinn, Douglas R. Galasko, Elaine R. Peskind

**Affiliations:** 1 Geriatric Research, Education, and Clinical Center, VA Puget Sound Health Care System, 1660 S. Columbian Way, Seattle, WA 98108 USA; 2 Department of Psychiatry and Behavioral Sciences, University of Washington, 1959 NE Pacific St, Box 356560, Seattle, WA 98195 USA; 3 Northwest Network (VISN-20) Mental Illness Research, Education, and Clinical Center (MIRECC), VA Puget Sound Health Care System, 1660 S. Columbian Way, Seattle, WA 98108 USA; 4 BSR&D Program, VA Puget Sound Health Care System, 1660 S. Columbian Way, Seattle, WA 98108 USA; 5 Department of Pathology, University of Washington School of Medicine, 1959 NE Pacific St, Box 357470, Seattle, WA 98195 USA; 6 Department of Pathology, Stanford University, 300 Pasteur Drive, Lane 235, Stanford, CA 94305 USA; 7 Parkinson’s Disease Research, Education, and Clinical Care Center, Portland VA Medical Center, 3710 SW Veterans Hospital Rd, Portland, OR 97239 USA; 8 Department of Neurology, Oregon Health and Science University, 3181 SW Sam Jackson Park Rd, L226, Portland, OR 97239 USA; 9 Department of Neurosciences, University of California at San Diego, 9500 Gilman Drive, La Jolla, CA 92093 USA

**Keywords:** Alzheimer’s disease, Cerebrovascular disease, Cerebrospinal fluid, Biomarkers, Age, Gender, *APOE* genotype

## Abstract

**Background:**

This study sought to evaluate gender and *APOE* genotype-related differences in the concentrations of cerebrospinal fluid (CSF) biomarkers for Alzheimer’s disease (AD) and cerebrovascular injury across the life span of cognitively normal adults.

**Methods:**

CSF amyloid beta_1–42_ (Aβ_42_), phospho-tau-181 (p-tau_181_), and total tau were measured in 331 participants who were between the ages of 21 and 100. CSF E-selectin and vascular cell adhesion protein 1 (VCAM1) were measured in 249 participants who were between the ages of 50 and 100.

**Results:**

CSF total tau and p-tau_181_ increased with age over the adult life span (*p* < 0.01) with no gender differences in those increases. CSF Aβ_42_ concentration varied according to age, gender, and *APOE* genotype (interaction of age × gender × ε4, *p* = 0.047). CSF VCAM1, but not E-selectin, increased with age (*p* < 0.01), but both were elevated in men compared to women (*p* < 0.01).

**Conclusions:**

Female *APOE*-ε4 carriers appear at higher risk for AD after age 50. In contrast, men may experience a relatively higher rate of cerebrovascular injury in middle and early old age.

**Electronic supplementary material:**

The online version of this article (doi:10.1186/s13195-017-0271-9) contains supplementary material, which is available to authorized users.

## Background

The reasons for gender differences in the risk of developing Alzheimer’s disease (AD) or other dementias remain unclear. Epidemiological studies have shown that the incidence of AD increases steeply with age in both men and women, but the rate of increase diverges between genders in later ages: after age 90, the incidence levels off in men, but it continues to increase in women [1–3]. One possibility is that men have higher mortality rates from cardiovascular disease than women. Indeed, the selective survival of men with a healthier cardiovascular risk profile and lower propensity to dementia may partly explain the relatively higher risk for dementia and AD that is observed in women versus men in older ages [4].

Another potential mechanism for the increased incidence of AD in women over the age of 80 is the loss, after menopause, of the neuroprotective effect of estrogens [5, 6]. The challenge in understanding the potential role of estrogen in AD is the long lag time between the drastic hormonal changes and clinical manifestations of AD. The critical period of hormonal changes in women is around age 50, which is 20–30 years prior to the clinical manifestation of dementia. Recent studies have shown that cerebrospinal fluid (CSF) biomarkers of neurodegenerative processes in AD (e.g., concentrations of amyloid beta_1– 42_ (Aβ_42_), total tau, phospho-tau-181 (p-tau_181_), and the tau/Aβ_42_ ratio) may reflect underlying AD pathophysiology long before changes in memory and cognition are clinically detectable [7, 8]. Decreases in the concentration of CSF Aβ_42_ (which correspond to increased deposition of amyloid in the brain [9]), increased p-tau_181_, and increased total tau occur up to 25 years prior to clinical expression [10]. Recently, we have shown that CSF E-selectin is a candidate biomarker for cerebrovascular injury [11]. E-selectin is an adhesion molecule produced by endothelial cells [12], usually after vascular injury, and is induced by cytokines [13, 14]. When released, E-selectin recruits leukocytes to sites of inflammation or injury [12], thereby contributing to cerebrovascular disease [13, 14]. Using these kinds of biomarkers as markers for preclinical AD or cerebrovascular injury, we may be able to bring our understanding and recognition of pathological processes closer to the time in life at which hormonal changes occur. In addition, each specific biomarker enables us to identify specific pathological processes of AD and other conditions, such as cerebrovascular disease. These pathological processes are commonly comorbid in older persons, affect cognitive function in different ways, and are difficult to tease apart clinically.

In the present study, we examined age-related gender differences in CSF biomarkers for AD (Aβ_42_, p-tau_181_, and total tau) and vascular injury (E-selectin and vascular cell adhesion protein 1 (VCAM1)) across the life span of cognitively normal adults. We also examined how these differences varied by apolipoprotein E (*APOE*) genotype. The findings from this study are important in elucidating various biological pathways that may play a role in the development of dementia and that may thereby inform the design of targeted prevention strategies.

## Methods

### Participants

Two cohorts of study participants were initially recruited for this study, one cohort for a biomarker study of AD and one cohort for a vascular biomarker study. The collection and analysis of CSF AD biomarkers and clinical data in the first cohort were approved by the University of Washington (UW) institutional review board (IRB; under approval numbers 01-8926-V and 01173). This cohort included 331 participants who ranged in age from 21 to 100 and who were enrolled at the UW Alzheimer’s Disease Research Center (ADRC) and five collaborating centers, including the University of California at San Diego (UCSD), Oregon Health & Science University (OHSU), Indiana University, University of Pennsylvania, and University of California at Davis. The collection and analysis of novel vascular biomarkers in the CSF of the second cohort occurred at three of these same institutions under the approval of their respective IRBs (UW 01-8926-V and 01173, UCSD 080012, and OHSU 6845). This cohort included 249 participants who ranged in age from 50 to 100. There was an overlap of 126 participants between the two cohorts, but these overlapping participants were all age ≥ 50. Additional file 1: Table S1 compares the characteristics of a subset of participants from the first cohort who are age ≥ 50 to the participants of the second cohort (who are all age ≥ 50).

All participants from both cohorts provided written informed consent prior to enrollment in the study. All participants also underwent standardized diagnostic evaluation as described in a previous report [15], were medically stable, and had no evidence or history of cognitive or functional decline. Mild cognitive impairments and dementia were ruled out in participants based on extensive neuropsychological testing, collateral information, and clinical diagnosis.

### Measurements of CSF biomarkers

CSF was obtained using 24-gauge Sprotte atraumatic spinal needles; the lumbar puncture procedures have been described elsewhere [16]. All CSF samples were analyzed at the UW ADRC laboratory using 0.5 ml aliquots that had been stored continuously frozen at −80 °C in polypropylene cryotubes and that had never previously thawed. The CSF samples were analyzed for Aβ_42_, p-tau_181_, and total tau using multiplex reagents (Luminex; InnoGenetics) according to the manufacturer’s instructions and as described previously [17]. CSF E-selectin and VCAM1 were measured using the Human Premixed Multi-Analyte Kit (R&D Systems, Minneapolis, MN, USA) [18]. Assays were performed following the manufacturer’s protocol with slight modifications (http://www.rndsystems.com/pdf/LXSAH.pdf) using a LiquiChip Luminex 200™ Workstation (Qiagen, Valencia, CA, USA). The *APOE* (M12529; GenBank) genotype was determined using a restriction digest method [19].

### Statistical analysis

The average difference in CSF biomarker concentration (Aβ_42_, total tau, p-tau_181_, E-selectin, and VCAM1) by gender was examined using linear regression. For these analyses, the CSF biomarker of interest was the dependent variable, and age, gender, and *APOE* genotype (i.e., presence versus absence of the *APOE*-ε4 allele) were the independent covariates; age was modeled as a three-degree restricted cubic spline to allow for nonlinear associations between age and CSF biomarker, with knots placed at the 5th, 50th, and 95th percentiles (corresponding to ages 24, 60, and 81 for the CSF AD biomarker cohort and ages 55, 70, and 85 for the CSF vascular biomarker cohort) [20]. The role of gender as an effect modifier in the association between CSF biomarkers and age was tested by adding an age × gender interaction term to the regression model. The additional role of the *APOE* genotype as an effect modifier in the association between CSF biomarkers, age, and gender was tested by adding a three-way age × gender × *APOE* genotype interaction term. Hypothesis testing relating to E-selectin was carried out using log E-selectin to satisfy the homogeneity of variance assumption, and the results were back-transformed to the original units, except where noted.

In instances where gender significantly affected the age–CSF biomarker association, nonparametric bootstrap methods were used to calculate 95% confidence intervals (CIs) for the mean difference in CSF biomarker concentration by gender across the age span. We selected the bootstrap approach for its ease in estimating CIs from complex models. This bootstrapping method generated 5000 datasets, and on each dataset the regression model for the CSF biomarker of interest was used to estimate a set of age-dependent mean differences in CSF biomarker by gender. The adjusted percentile method was then used in these 5000 sets of differences to calculate 95% CIs for the mean difference in biomarker by gender across the age span [21]. Linear regression was also used to determine the association between CSF vascular biomarkers (the dependent variables) and vascular risk factors (body mass index (BMI), history of diabetes mellitus, and hypertension or coronary artery disease), adjusting for age and gender. BMI and age were both modeled as three-degree restricted cubic splines as stated earlier. The association between CSF E-selectin and CSF VCAM1 was assessed using linear regression with CSF VCAM1 as the dependent variable and CSF E-selectin as the independent variable. Effect modification due to age or gender was assessed by testing the significance of the E-selectin by age or gender interaction term.

R 2.15.2 [22] and the *Hmisc* and *rms* packages [23] were used to carry out the regression models, and the *boot* package [24] was used to perform the bootstraps. Mean changes in biomarkers estimated from the regression models presented in the text are accompanied with 95% CIs in parentheses.

## Results

The demographic and CSF biomarker characteristics of the participants are presented in Table 1. Male participants in the AD biomarker cohort had significantly higher levels of education than female participants in the CSF AD biomarker cohort (*p* = 0.016), and male participants in the vascular biomarker cohort had lower MMSE scores than female participants in the vascular biomarker cohort (*p* < 0.01). However, there were no other significant gender differences in mean age, BMI, or *APOE*-ε4 status in either cohort.

**Table 1:** Characteristics of participants by gender

	CSF AD biomarker sample		CSF vascular biomarker sample	
	Female (*n* = 178)	Male (*n* = 153)	Female (*n* = 141)	Male (*n* = 108)
Age (years)	57.5 (16.8) (21–100)	56.0 (19.6) (22–88)	70.0 (8.5) (50–100)	72.1 (9.1) (50–90)
BMI (kg/m^2^)	25.8 (4.3) (18–41)	26.4 (3.4) (20–37)	25.7 (4.3) (18–39)	26.3 (3.0) (20–36)
Education (years)	15.8 (2.5) (10–22)	16.5 (2.8)* (10–27)	15.8 (2.5) (9–23)	16.2 (2.6) (10–25)
MMSE score	29.3 (0.9) (26–30)	29.2 (1.2) (25–30)	29.5 (0.9) (25–30)	28.8 (1.4)* (24–30)
*APOE-*ε4 positive, *n* (%)	67 (38)	48 (31)	49/136 (36)	33/100 (33)
Aβ_42_ (pg/ml)	331 (135) (63–821)	323 (116) (98–801)		
Total tau (pg/ml)	47.9 (13.4) (7–91)	50.5 (15.0) (18–130)		
p-tau_181_ (pg/ml)	31.3 (8.4) (<7–67)	32.6 (12.7) (9–107)		
E-selectin (pg/ml)			42.1 (33.2) (1.5–188)	60.3 (42.1) (1.5–226)
VCAM1 (ng/ml)			115 (59) (34–343)	147 (70) (42–429)

Data presented as mean (standard deviation) (range), unless stated otherwise

*A*β_*42*_ amyloid beta_1–42_, *AD* Alzheimer’s disease, *APOE* apolipoprotein E, *BMI* body mass index, *CSF* cerebrospinal fluid, *MMSE* Mini-Mental State Examination, *p-tau*_*181*_ phospho-tau-181, *VCAM1* vascular cell adhesion protein 1

*p < 0.05 for gender difference within each cohort by *t* test or χ^2^ test

### Gender differences in CSF AD biomarker concentrations across the life span

**Fig. 1:**
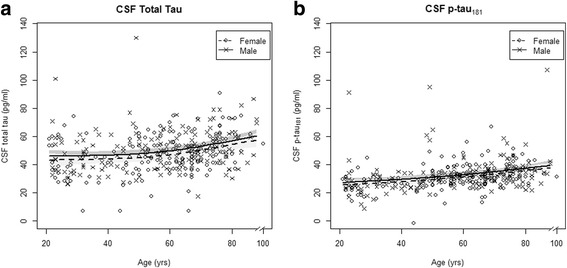
CSF AD tau biomarkers by age and gender. Scatterplot of CSF total tau (**a**) and p-tau_181_ (**b**) vs participant age and predicted means with 95% CIs from linear regression on gender × age interaction, adjusted for *APOE* genotype. *CSF* cerebrospinal fluid, *p-tau*_*181*_ phospho-tau-181, *yrs* years

Mean CSF total-tau and p-tau_181_ concentrations were significantly increased (*p* < 0.01) with increasing participant age in both men and women (see Fig. 1a, b). There were no differences in average total tau or p-tau_181_ by gender (Table 2). Furthermore, there was no evidence that the increase in total tau or p-tau_181_ across age differed by gender (age × gender interaction, *p* ≥ 0.43) or that differences in age-adjusted total tau or p-tau_181_ by gender were further modified by *APOE* genotype (age × gender × *APOE* genotype interaction, *p* ≥ 0.16; Table 2).

**Table 2:** Changes in CSF biomarkers by age and gender and interactive effects predicted from regression models

			*p* value for interaction term^a^	
	Age difference^b^	Gender difference ^c^	Age × gender	Age × gender × ε4
Aβ_42_ (pg/ml)	37 (–2, 76), 0.09	–5 (–32, 23), 0.75	0.51	0.047
Total tau (pg/ml)	8.9 (4.7, 13.1), <0.01	2.7 (–0.3, 5.7), 0.08	0.43	0.21
p-tau_181_ (pg/ml)	8.5 (5.4, 11.6), <0.01	1.6 (–0.6, 3.8), 0.15	0.85	0.16
E-selectin (pg/ml)^d^	13 (–6, 43), 0.47	20 (10, 33), <0.01	0.04	0.65
VCAM1 (ng/dl)	66 (30, 102), <0. 01	24 (8, 39), <0.01	0.34	0.64

*A*β_*42*_ amyloid beta_1–42_, *APOE* apolipoprotein E, *CSF* cerebrospinal fluid, *p-tau*_*181*_ phospho-tau-181, *VCAM1* vascular cell adhesion protein 1

^a^From linear regression models of CSF biomarker on age × gender interaction adjusted for *APOE*-ε4 status or on age × gender × *APOE*-ε4 status interaction

^b^Age difference: mean difference (95% confidence interval) and *p *value in CSF Aβ_42_, total tau, and p-tau_181_ for an increase in participant age from 25 to 75 years; and mean difference and *p* value in E-selectin and VCAM1 for an increase in participant age from 50 to 75 years

^c^Gender difference: mean difference (95% confidence interval) and *p *value in concentration of CSF biomarker in males minus females

^d^Hypothesis testing carried out on log E-selectin, with results presented back-transformed into the original scale

**Fig. 2:**
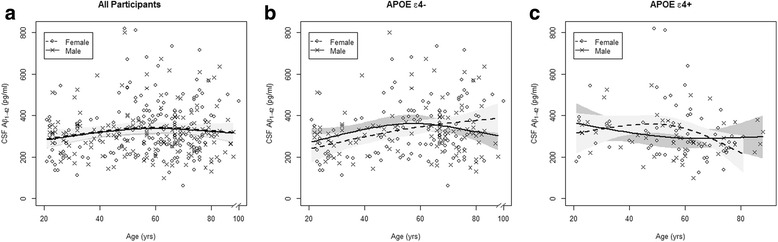
CSF Aβ_42_ by age and gender and stratified by *APOE* genotype. Scatterplot of CSF Aβ_42_ vs participant age and predicted means with 95% CIs predicted from linear regression on gender × age interaction adjusted for *APOE-*ε4 status (**a**), and stratified by *APOE*-ε4 negative (**b**) and positive (**c**) from linear regression on gender by age × *APOE-*ε4 interaction. *A*β_*42*_ amyloid beta_1–42_, *APOE* apolipoprotein E, *CSF* cerebrospinal fluid, *yrs* years

In contrast to the age–CSF tau relationships, there was a lack of overall association between CSF Aβ_42_ and age or between CSF Aβ_42_ and gender (Table 2, Fig. 2a). The association between CSF Aβ_42_ and age was complex due to the effect modification by gender and *APOE* genotype (age × gender × *APOE* genotype interaction, *p* = 0.047) and the presence of nonlinear trends in CSF Aβ_42_ across the age span (Fig. 2, Additional file 1: Table S2). Specifically, the age-related and gender-related differences in CSF Aβ_42_ were dependent on *APOE* genotype. In the ε4 noncarriers, average CSF Aβ_42_ increased monotonically across participant age for females, whereas CSF Aβ_42_ increased up to midlife and then leveled off for males (Fig. 2b). In the ε4 carriers, average CSF Aβ_42_ decreased gradually across participant age in males up to midlife and then leveled off. By contrast, in females, average CSF Aβ_42_ remained relatively high through age 50 and then had a rapid decline after age 50 (Fig. 2c). For example, mean decrease in CSF Aβ_42_ from age 50 to age 75 was 103 pg/ml in female ε4 carriers but only 9 pg/ml for male ε4 carriers. The magnitudes of predicted mean change in CSF Aβ_42_ along different age intervals and stratified by gender and *APOE* are presented in Additional file 1: Table S2.

### Gender differences in CSF vascular biomarker concentrations across the life span

**Fig. 3:**
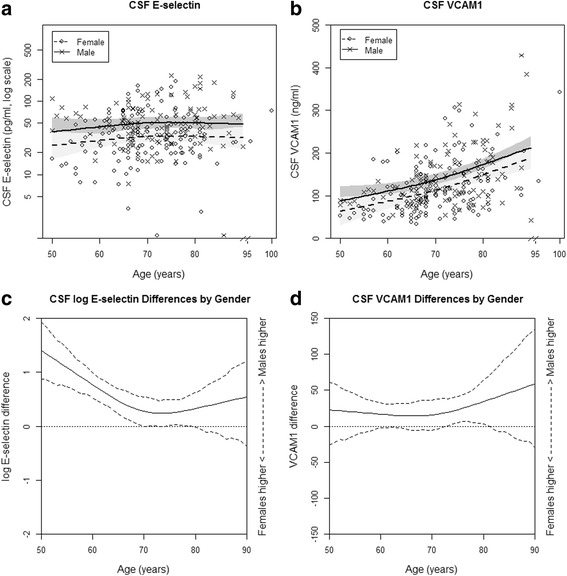
CSF vascular biomarkers by age and gender. Scatterplot of CSF E-selectin, log scale (**a**) and VCAM1 (**b**) vs participant age and predicted means with 95% CIs, and mean male minus female difference in log E-selectin (**c**) and VCAM1 (**d**) with 95% CIs predicted from linear regression on gender × age interaction, adjusted for *APOE* genotype. *CSF* cerebrospinal fluid, VCAM1 vascular cell adhesion protein 1

Mean CSF E-selectin concentration was not correlated with participant age (*p* = 0.47; Table 1, Fig. 3a), whereas mean VCAM1 concentration increased with participant age (*p* < 0.01; Table 1, Fig. 3b). Males had higher mean CSF E-selectin and VCAM1 levels than females (*p* < 0.01; Table 1). Furthermore, gender was a significant effect modifier in the association between E-selectin and participant age (age × gender interaction, *p* = 0.03; Table 2, Fig. 3a, c) such that males had higher mean E-selectin than females at earlier participant ages (i.e., approximately age 50–70) but not at older ages. There was no effect modification of *APOE* genotype on the age-dependent gender difference in E-selectin (age × gender × *APOE*-ε4 status, *p* = 0.65), and there was no evidence that gender modified the relationship between VCAM1 and age (*p* = 0.35; Fig. 3b, d) or that age-dependent gender differences in VCAM1 were modified by *APOE* genotype (age × gender × *APOE*-ε4 interaction, *p* = 0.64). As a sensitivity analysis, we repeated the previous analyses in participants who took part in both the AD and vascular biomarker study (*n* = 126). In these analyses, males still had higher mean E-selectin levels than females (*p* = 0.052). The effect modification pattern of gender on the age–E-selectin relationship was also similar, but the age × gender interaction was no longer statistically significant (*p* = 0.13). As in our primary analysis, males also had higher mean VCAM1 levels than females (*p* = 0.02).

### Relationship of CSF E-selectin or VCAM1 with vascular risk factors

There were 55 participants with hypertension, 12 with diabetes mellitus, and 11 with coronary artery disease in the CSF vascular subsample. CSF E-selectin concentration was correlated positively with BMI after adjusting for age and gender (*p* < 0.01). Mean CSF E-selectin was higher in participants with diabetes mellitus by 19 pg/ml (95% CI: –2, 50) but the relationship did not reach statistical significance after adjusting for age and gender (*p* = 0.08). VCAM1 was not significantly correlated with BMI (*p* = 0.71) but was elevated in participants with diabetes mellitus by 61 ng/ml (95% CI: 28, 95, *p* < 0.01). There was no significant association between E-selectin or VCAM1 and history of hypertension or coronary artery disease (*p* > 0.05). CSF E-selection and VCAM1 was positively correlated such that *r* = 0.23 (*r* ^2^ = 0.05, linear regression *p* < 0.01). This correlation was not modified by age or gender (CSF E-selectin by age or gender interactions *p* > 0.67).

## Discussion

In this study of cognitively normal adults, we found that the CSF AD biomarkers total tau and p-tau_181_ increased with participant age over the adult life span (i.e., ages 20–100) with no significant gender or *APOE* genotype differences in those increases. In contrast, CSF Aβ_42_ concentration varied according to age, gender, and *APOE* genotype.

The nonlinear relationship between age and CSF Aβ_42_ is likely because our sample includes both participants who are experiencing normal aging as well as participants over the age of 50 who may have preclinical AD. Although the process of brain amyloid metabolism is not entirely clear in normal aging, during the early stages of AD brain Aβ_42_ starts to aggregate, causing a “sink” effect characterized by a decrease in Aβ_42_ concentration in the CSF [9, 25]. Likewise, we reported previously that CSF Aβ_42_ decreases with age in cognitively normal adults across the life span (ages 21–88), with reductions in CSF Aβ_42_ becoming apparent at earlier ages for *APOE-*ε4 carriers than ε4 noncarriers [26]. This is consistent with the report of low CSF Aβ_42_ and continuous decline in middle-aged *APOE*-ε4 carriers [27], as well as the well-established link between *APOE* ε4 and an increased risk of β-amyloidosis [9, 28, 29].

The present study extends these findings by demonstrating more complex three-way interactions of age × gender × *APOE* genotype and different patterns of age-related change of CSF Aβ_42_ in female versus male carriers of *APOE* ε4. Male *APOE*-ε4 carriers exhibited modest average decline in CSF Aβ_42_ concentrations across participant age, whereas female *APOE*-ε4 carriers exhibited a steeper average decline in CSF Aβ_42_ concentration with increasing participant age starting around age 50, coincident with the onset of menopause. Whether female *APOE*-ε4 carriers become more vulnerable to AD after the onset of menopause should be investigated further. By contrast, in female *APOE*-ε4 noncarriers, CSF Aβ_42_ levels did not drop around age 50 and, instead, steadily increased over the life span. It is plausible that brain amyloidosis increases with aging; although no studies have investigated this in humans, a recent study of brain Aβ_42_ in rhesus monkeys demonstrates that Aβ_42_ increases with aging in both brain and CSF [30]. It is also possible that the observed age-related changes are partly due to age-related alterations in the clearance of Aβ_42_ in brain or age-related changes in CSF flow. Because our current study uses a cross-sectional design, future studies that employ a longitudinal design will be necessary to clarify the nature of age-related Aβ_42_ metabolism in the brain.

Gender differences in the brain aging and disease processes of AD are not well understood, and the existing literature in this area is inconsistent [8, 28, 31, 32]. Using the Alzheimer’s Disease Neuroimaging Initiative (ADNI) dataset, Altman et al*.* [32] reported that average levels of CSF total tau and tau/Aβ_42_ ratio in participants with mild cognitive impairment (MCI) were higher in female *APOE-*ε4 carriers than male *APOE-*ε4 carriers; but in cognitively normal controls, they found no gender difference in either CSF Aβ_42_ or total-tau concentration, regardless of *APOE* genotype. Consistent with the findings in the normal controls, a later analysis of the ADNI dataset showed no modifying effects of APOE × gender on CSF biomarkers, but this analysis reports a significant interactive effect of *APOE* ε4 × gender on brain metabolism and structure, in that female *APOE-*ε4 carriers had greater hypometabolism and atrophy than female ε4 noncarriers or male ε4 carriers [33]. However, a study by Jack et al*.* [28] of neuroimaging biomarkers in cognitively normal controls who were over the age of 60 showed that men had poorer memory and smaller hippocampal volume than women; they also found that memory and hippocampal volume decline began at earlier ages than brain Aβ deposition. The authors speculated that this earlier decline in hippocampal volume may be due to cerebrovascular disease and age-related tauopathy. Our findings of a higher mean CSF concentration of vascular biomarkers in men compared to women, especially in E-selectin from age 50 to 70, but no gender differences in total tau or p-tau_181_ suggest that the reduction in hippocampal volume observed in the Jack et al. study may be the result of cerebrovascular disease rather than age-related tauopathy. This is consistent with the expectation that men are at higher risk for cerebrovascular disease than women, especially in middle age and early old age [4].

There is increasing evidence that small-vessel brain disease, especially when characterized by microinfarcts, plays an important role in the cognitive health of older persons [34, 35], particularly older persons with hypertension and diabetes mellitus [36, 37]. Serum E-selectin, an endothelial–leukocyte adhesion molecule, plays an important role in inflammation. It is elevated in metabolic syndrome [14, 38], in overweight women with additional cardiovascular risk factors [38], in persons with diabetes mellitus who have silent cerebral ischemic infarcts [14], and in diabetes mellitus patients in whom these cerebral ischemic lesions have progressed further [13]. Serum E-selectin is also linked to cerebral microbleeds [39]. In our earlier study, CSF E-selectin was found to be highly correlated with BMI and diabetes mellitus in persons with a diagnosis of AD; it was also elevated in clinically diagnosed AD patients who had no AD CSF biomarker signature [11]. In the present study, the correlation between CSF E-selectin and BMI, as well as the elevation in concentration of CSF VCAM1 in cognitively normal participants with diabetes mellitus, suggests that CSF E-selectin and VCAM1 may be biomarkers for cerebrovascular injuries in the brain. CSF E-selectin and the VCAM1 concentration in CSF were correlated modestly, but their relationship to age and gender were somewhat different. That is, VCAM1 increased with participant age in both genders, although males had higher mean VCAM1 levels than females across the age span of 50–100 years. In contrast, E-selectin did not increase with participant age, but males did have higher mean E-selectin levels than females aged between 50 and 70 years. Neuroimaging and/or autopsy studies are necessary to determine whether these two potential vascular biomarkers indicate different pathological processes in the development of cerebrovascular disease. Further studies are also necessary to determine whether elevated CSF E-selectin and VCAM1 are associated with functional impairments, especially to determine whether higher levels of E-selectin in men may explain their poor cognitive performance as observed in studies by Jack et al*.* [28] and our earlier study [15]. Such studies would also help to explain recent observations of weaker associations between cognitive function or hippocampal volume and CSF AD biomarkers of Aβ_42_ and tau in men than in women [40], because cognitive impairments in men may be due in part to vascular damage that has been underestimated.

Our study has a few limitations that may affect the interpretation of the results. First, because this is a cross-sectional study, our observations of age-related change are based on adults of different ages examined at a single time point rather than over time. The lower average concentration in the women with APOE ε4 after age 50 may reflect a high proportion of preclinical AD cases with lower CSF Aβ_42_ concentration rather than the age-related decline. The age-related longitudinal changes in CSF concentration of Aβ_42_, total tau, and p-tau_181_ in individual male and female adults may differ from those exhibited by a sample of adults of different ages who were measured cross-sectionally. Although a longitudinal study of these changes would be optimal, a study that spans 80 years, from age 20 to age 100, is not feasible. Second, the differences in CSF Aβ_42_ change across age by gender and *APOE* genotype were accompanied by relatively large variability due to the stratification of our sample into gender and *APOE* genotype subgroups. Confirmation of these findings in a larger sample should be carried out. Finally, we were unable to measure both AD and vascular biomarkers in the same cohort, and the cohort for our vascular biomarker study included only middle-aged and older participants. Because the effects of gender and *APOE* genotype on each biomarker are age dependent, a study that includes younger participants may yield different findings from the age and gender differences in E-selectin and VCAM1 observed in the current study. Thus, further study is needed of these biomarkers across the entire adult age span. Despite these limitations, this is one of the first studies of cognitively normal adults to demonstrate age-related gender differences in both CSF AD and vascular biomarkers across a large age span with an interactive effect of *APOE* genotype.

## Conclusions

The AD pathophysiologic process over the adult life span is complex, and brain β-amyloidosis is dependent on age, gender, and *APOE* genotype. Female ε4 carriers appear at higher risk for AD after menopause, whereas men may experience a relatively higher rate of cerebrovascular injury, especially in middle and early old age, which may contribute to poorer cognitive performance.

## Additional file

Additional file 1: Tables S1 and S2. Table S1 presents participant characteristics and Table S2 presents mean change in CSF Aβ_42_ by increase in age and stratified by gender and *APOE* genotype predicted from linear regression of CSF Aβ_42_ on the three way age × gender × *APOE* genotype interaction. (DOCX 14 kb)

## Abbreviations

AD: Alzheimer’s disease
ADNI: Alzheimer’s Disease Neuroimaging Initiative
ADRC: Alzheimer’s Disease Research Center
*APOE*: Apolipoprotein E
Aβ_42_: Amyloid beta 42
BMI: Body mass index
CI: Confidence interval
CSF: Cerebrospinal fluid
MCI: Mild cognitive impairment
p-tau_181_: Phospho-tau-181
VCAM1: Vascular cell adhesion protein 1
