# Supplementary material for: Cerebrospinal fluid biomarkers for Alzheimer's and vascular disease vary by age, gender, and APOE genotype in cognitively normal adults
Source: Alzheimers Res Ther. 2017 Jul 3;9:48. doi: 10.1186/s13195-017-0271-9 (PMC5496132; doi:10.1186/s13195-017-0271-9)
Supplement: Additional file 1: Tables S1 and S2. — Table S1 presents participant characteristics and Table S2 presents mean change in CSF Aβ42 by increase in age and stratified by gender and APOE genotype predicted from linear regression of CSF Aβ42 on the three way age × gender × APOE genotype interaction. (DOCX 14 kb) [file 13195_2017_271_MOESM1_ESM.docx]

| Mean [SD], (range)  or N (%) | **Cohort 1**  **age ≥ 50 years**  n=229 | **Cohort 2**  n=249 | **Participants in both cohorts**  n = 126 |
| --- | --- | --- | --- |
| Age, years | 67.0 [9.9], 50–100 | 70.1 [8.8], 50–100 | 71.1 [8.4], 50–100 |
| BMI (kg/m^2^) | 26.1 [3.7], 18–40 | 26.0 [3.8], 18–39 | 26.0 [3.2], 19–34 |
| Education, years | 16.0 [2.7], 10–25 | 16.0 [2.5], 9–25 | 16.1 [2.4], 10–25 |
| MMSE score | 29.2 [1.0], 25–30 | 29.2 [1.2], 24–30 | 29.2 [1.1], 25–30 |
| Sex, male, n (%) | 96 (42%) | 108 (43%) | 60 (48%) |
| *APOE-*ε4+, n (%) | 82 (36%) | 82 (35%) | 42 (33%) |
| Diabetes mellitus, n (%) | 2 (1%) | 12 (5%) | 1 (1%) |
| Hypertension, n (%) | 30 (13%) | 55 (22%) | 14 (11%) |
| Coronary artery disease, n (%) | 1 (<1%) | 11 (4%) | 0 (0%) |
| BMI: body mass index; MMSE: Mini-Mental State Examination; SD: standard deviation. | | | |

Additional file 1

**Table S1**. Characteristics of participants aged 50+ in Cohort 1 and of all participants in the Cohort 2 participants

**Table S2**. Mean change in CSF Aβ_42_ by increase in age and stratified by gender and *APOE* genotype predicted from linear regression of CSF Aβ_42_ on the three way age x gender x *APOE* genotype interaction

| ***APOE* genotype** | **Gender** | **Mean CSF Aβ_42_ [95%CI] changes**  **by age increase, years** | | |
| --- | --- | --- | --- | --- |
|  |  | **25 to 50** | **50 to 75** | **25 to 75** |
| ε*4* - | Male | 69 [0, 138] | -20 [-70, 30] | 49 [-15, 112] |
| ε*4* - | Female | 70 [8, 133] | 46 [-3, 95] | 117 [49, 183] |
| ε*4* + | Male | -52 [-144, 40] | -9 [-78, 60] | -62 [-162, 39] |
| ε*4* + | Female | 33 [-53, 120] | -103 [-184, -22] | -69 [-168, 30] |
